# Diet and feeding strategy of Northeast Atlantic mackerel (*Scombrus scomber*) in Icelandic waters

**DOI:** 10.1371/journal.pone.0225552

**Published:** 2019-12-30

**Authors:** Cecilia Kvaavik, Gudmundur J. Óskarsson, Anna Kristín Daníelsdóttir, Gudrún Marteinsdóttir

**Affiliations:** 1 Pelagic Division, Marine and Freshwater Research Institute, Skulagata, Reykjavik, Iceland; 2 Institute of Biology, University of Iceland, Sturlugata, Reykjavik, Iceland; 3 Matis, Vinlandsleið, Reykjavik, Iceland; Havforskningsinstituttet, NORWAY

## Abstract

Predator-prey relations, as well as the trophic ecology of highly migratory marine species, is important to understand their impact on the ecosystem. Conventional methods were used to study the diet composition and feeding strategy of the Northeast Atlantic mackerel (*Scombrus scomber*), during their summer feeding migration to Icelandic waters in 2009–2014. In addition, generalised additive modelling (GAM) was used to determine which biological and environmental factors contribute to the variation of their stomach weight in the years 2011–2014. From the dietary analysis, we found that calanoid copepods (especially *Calanus finmarchicus*) were the most important contributor to the overall diet of mackerel in the years studied. Although in some years and areas, they also preyed heavily on larger prey items such as euphausiids, amphipods and megalopa larvae of crab and shrimp. The GAM showed that temperature and the time the day of sampling were significant explanatory variables for the stomach weight, while zooplankton biomass did not seem to have much influence. The Northeast Atlantic mackerel are ferocious feeders upon copepods, as well as exhibiting an overall opportunistic feeding strategy. During their feeding migration in Icelandic waters, they were found to feed on the most dominant species available to them.

## Introduction

Marine ecosystems are under an increasing threat from climate changes on top of environmental variability. Highly migratory pelagic fish species occupying large and different marine ecosystems might respond to such changes by altering migration patterns, distribution and feeding habits. These responses will impact the ecosystems inhabited through predator-prey interactions, which can be difficult to predict and observe [1,2]. Hence, understanding these impacts requires studying fish diets and feeding habits (i.e. prey selection and specialisation). Such studies provide the basis for understanding trophic interactions in marine food webs and the effect the predator has on the ecosystem as a whole [3,4] and are crucial for more ecosystem-based fishery management.

Recent warming of sea surface temperatures around Iceland has opened up new habitats for more migratory temperate species, such as the Northeast Atlantic mackerel (*Scombrus scomber*) [5–7]. The reason for the expansion of the Northeast Atlantic mackerel (hereafter called “mackerel”) feeding migration in the last decade to the north and northwest is not evident. However, this migration is postulated to be the result of many co-contributing factors such as; increased stock size (170% from 2002–2013 [8]), gradual increase in temperature [5], decline in zooplankton biomass in the Norwegian Sea [9] and possible competition with other major pelagic fish stocks such as herring (*Clupea harengus*) [5,8]. This expansion has meant that mackerel are now found in large numbers within Icelandic waters during the summer [10–12]. At this time of year, the surface water is above 7°C in Icelandic waters, which seems to restrict the mackerel distribution during its summer feeding migration, as they prefer water temperatures within the range 9–13°C [10,12–15].

Studying the diet composition of mackerel in the marine ecosystem around Iceland is crucial for understanding its position and trophic interactions in the marine food web and how it might differ from other ecosystems. Information on mackerel feeding ecology, provides much-needed information on mackerel growth conditions, feeding competition and distributional shifts [8,16,17]. Mackerel has proven to be a very ferocious predator and exhibits both particle and filter feeding [18,19], and while feeding in Icelandic waters during the summer, it has been reported to gain about 43% in body weight on average [20]. This example, signifying its massive feeding activity, together with the fact that the mackerel stock is one of the largest pelagic fish stocks in Northeast Atlantic [21] means that mackerel is a major component in the epi-pelagic ecosystems, including Icelandic waters [10]. Further knowledge on its feeding habits and diet composition is therefore highly relevant for a better understanding of the ecosystems’ functioning.

Previous stomach content analysis of mackerel in the Northwest Atlantic has shown that their diet consists, for the most part, of mesozooplankton (i.e. calanoid copepods, euphausiids and amphipods). Additionally, some studies have also shown mackerel to feed heavily on larger prey items such as juvenile fish (e.g. herring, sandeel *Ammodytes* spp. and capelin *Mallotus villosus*), fish eggs as well as larger crustaceans and squid [13,16,20,22–27]. A potential increase in predation of crustaceans, fish eggs and larvae by mackerel in Icelandic waters can have a detrimental effect on the survival of native populations of seabirds, marine mammals and fish, even to the point of affecting the recruitment rates of these species, who rely on these prey items as their primary food source [28–31]. It is therefore imperative to conduct more studies on the feeding habits and potential impact of mackerel in Icelandic waters and elsewhere.

The objective of this study is to examine the diet composition and feeding strategy (e.g. prey selectivity) of mackerel during their summer feeding in Icelandic waters in 2009–2014 through stomach content analysis. It includes examining the effects of predator size as well as temporal and spatial variation in stomach contents. This will be done by using generalised additive modelling (GAM) to estimate which environmental factors contribute to this variation. All this will provide more comprehensive knowledge on the biology and trophic ecology of this species and expand our understanding of the possible impact mackerel can have on similar native species in this ecosystem and elsewhere.

## Materials and methods

### Study area

**Fig 1:**
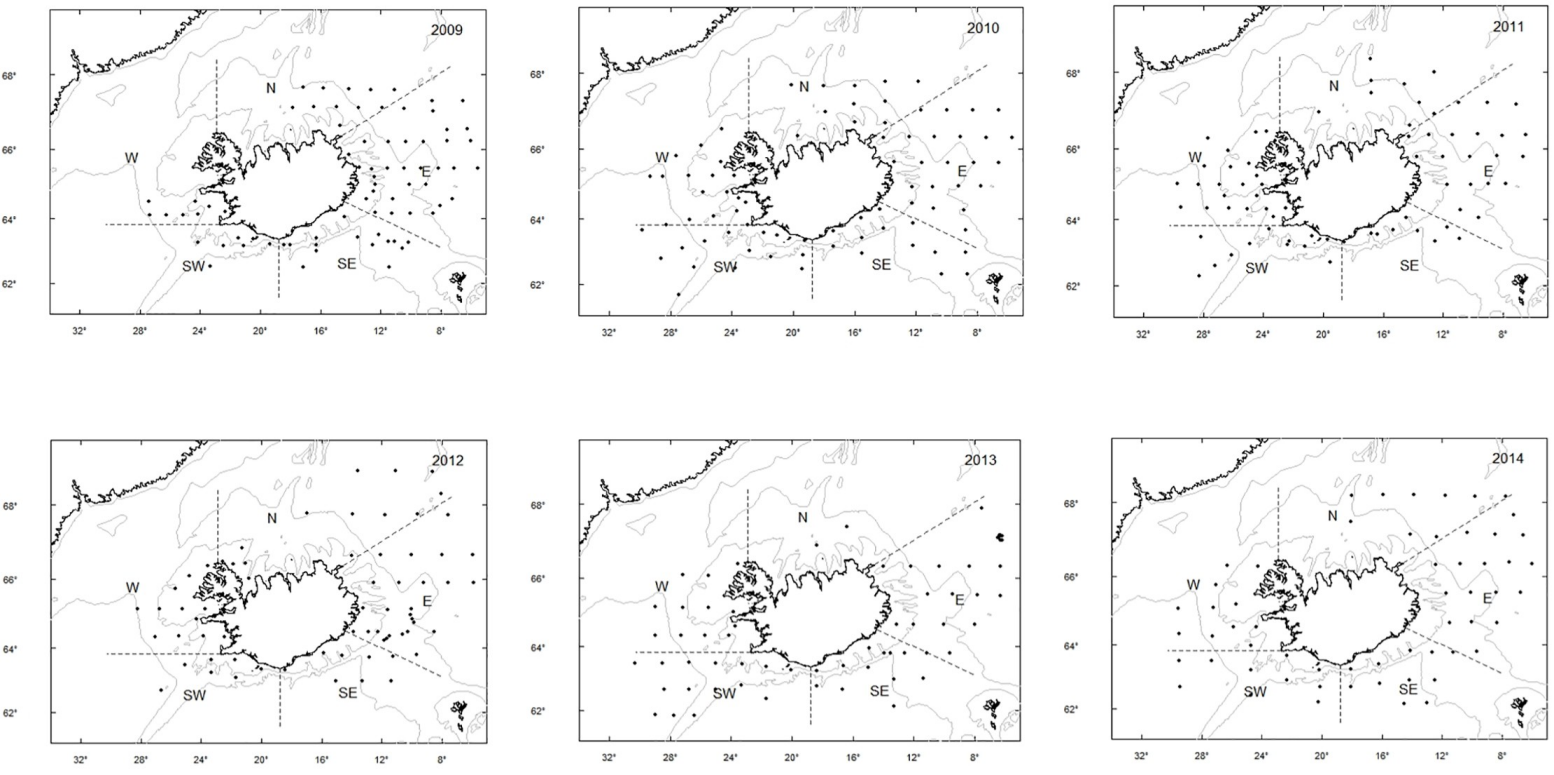
Study area. Sampling stations for Northeast Atlantic mackerel, plankton and CDT in Icelandic waters in 2009–2014, separated into five sub-areas: north (N), east (E), southeast (SE), southwest (SW) and west (W).

Iceland is situated where two submarine ridges meet, the Mid-Atlantic Ridge and the Greenland-Scotland Ridge, just below the Arctic Circle [32,33]. These ridges affect the flow of surface waters around Iceland, with more saline and warm Atlantic water flowing along the south, southwest and southeast coast following the North Atlantic—and Irminger Current and more fresh and cold Arctic water masses flowing towards the north and east coast originating from the East Iceland—and East Greenland Current [6,7,32]. Consequently, the area south and west of Iceland contains warm Atlantic water while colder mixed Atlantic and Arctic waters are north and east of Iceland. During the summers, warming of the surface waters creates a thermocline at around 30m depth, so the surface waters of the north- and east coast of Iceland can be above 7°C and are thereby at a suitable temperature range for mackerel [12,34–36]. The stomach sampling covered these different water masses around Iceland (Fig 1).

### Stomach sampling

Mackerel stomachs were collected in the International Ecosystem Summer Survey in Nordic Seas (IESSNS), which is coordinated by ICES (International Council for the Exploration of the Seas) and took place from July to August in 2009–2014 (Fig 1). During this survey, a total of 18601 mackerel were collected for further analysis in Icelandic waters, ranging from 18–46 cm in length (S1 Table and S1 Fig). Of those, 3777 stomachs were sampled for dietary analysis. In 2009 and 2010 the samples were taken in an epipelagic trawl, whereas from 2011 and onwards a specially designed pelagic Multpelt 832 trawl was used. The trawl hauls were taken in the surface waters at predefined locations around Iceland [11]. The vertical opening of the trawls varied from 16.5m (2009 and 2010) to 30–35 m (from 2011 onwards) [10]. Trawl catches were sorted and weighed, and the fish were identified to species level and other taxa to higher taxonomic levels. Total length (L; 1.0 cm), whole body weight (W; 0.1 g), weight of gonads (0.1 g), sex, maturity stage and age were recorded for all mackerel. Where possible, ten mackerel stomachs were collected from each station, and the stomach weight (including the stomach content) was recorded (0.1 g) and frozen immediately to ₋20°C for later analyses ashore. In the laboratory, the stomach content from individual fish was analysed by use of a microscope and the prey items were grouped into the lowest taxonomic group, excluding parasitic animals and unidentified objects (these were weighed but not counted). Small prey individuals within a taxonomic group were counted and weighed together (wet weight to nearest 0.01 g) and for larger prey items (i.e. fish) the total length was also measured (mm). For stomachs containing large amounts of zooplankton, a sub-sample was taken, by mixing the sample and removing 1-2mL of the mixture to a counting chamber with a pipette. Prey items that were much digested (e.g. fish otoliths or euphausiid eyes) were all identified to the lowest taxonomic level.

The total length of mackerel examined ranged from 19-48cm. To evaluate variation in food habits as a function of predator size, mackerel were divided into three length groups (≥33cm (S); 34-38cm (M); ≤39 cm (L)), with a minimum of 50 fish in each group. A separation into five sub-areas (south, southeast, southwest, west and north) within Icelandic waters (Fig 1) was done to examine if there was a difference in diet composition between these areas. So, within each area, individual mackerel stomachs from all stations were pooled. This separation was based on oceanographic boundaries (SE vs. E and W vs. N; see description of study area above) and more arbitrary boundaries related to geographical features (W vs. SW), migration distance for mackerel coming from southeast (N vs. E and W vs. SW), size of the continental shelf and size of the areas (SE vs. SW) [20,37,38].

Our study did not involve any endangered or protected species and no experimentation with live animals was performed. No other ethical issues applied to the present research project. Special permissions or rules for sacrificing fish from an animal ethics committee, are at present non-existing in Iceland for the scientific sampling of fish. Usually, trawling and handling of the fish onboard the vessels, including sorting and sampling leads to high mortality. Therefore the fish collected as part of this research were killed as rapidly as possible. Hence, all the fish were dead before any surgical procedure occurred.

### Environmental and ecological measurements

**Fig 2:**
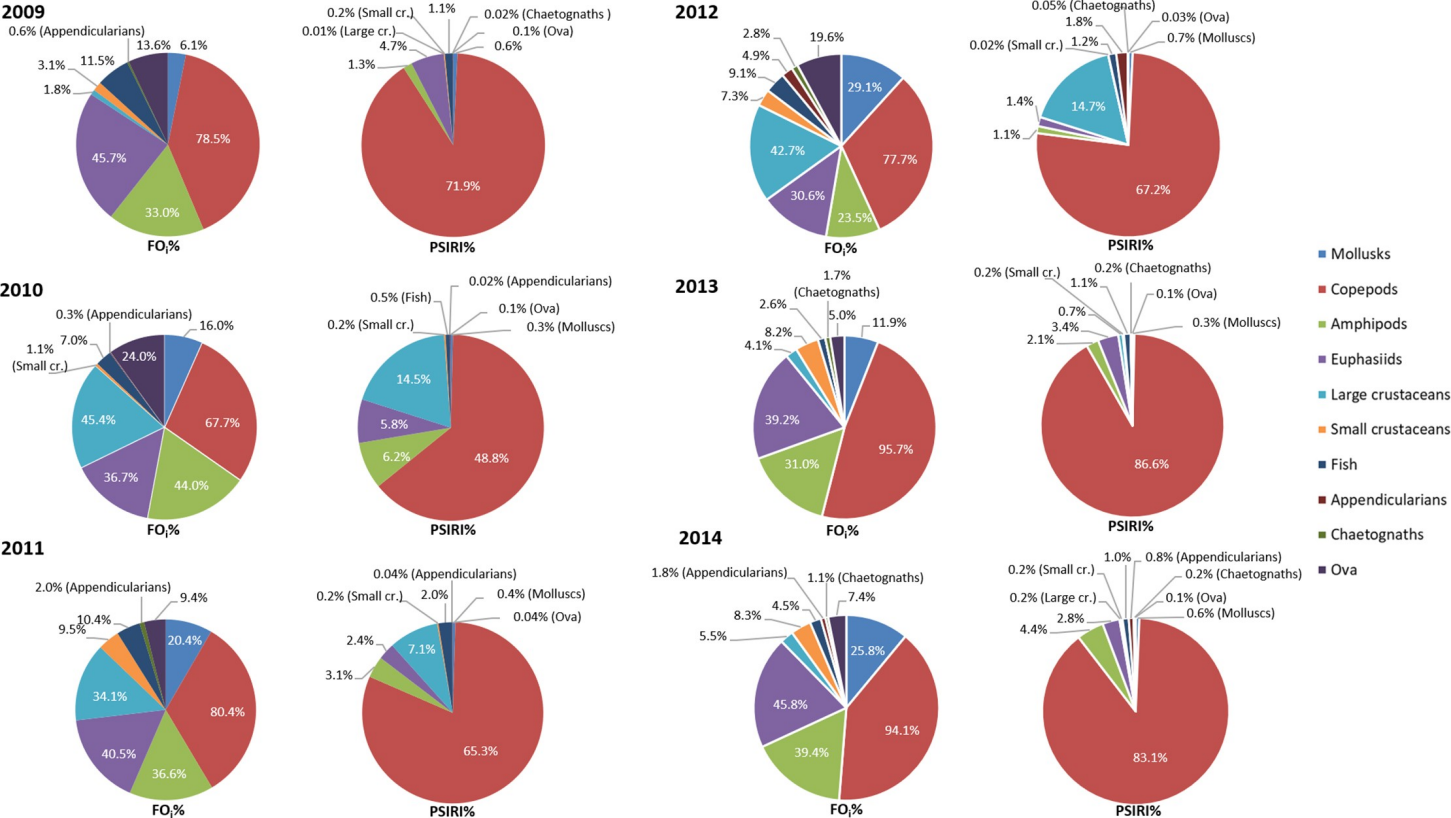
Frequency of Occurrence (FOi%) and Prey-Specific Index of Relative Importance (PSIRI%) for different prey groups of Northeast Atlantic mackerel in Icelandic waters in the years 2009–2014.

Zooplankton were sampled during the IESSNS survey in 2009–2014 using a WP2-net with a mesh size of 200μm at the same locations as the trawl hauls were taken. The nets were hauled vertically from a depth of 200m, or from the near bottom at shallower stations, to the surface at a speed of 0.5 m/s. All samples were split in two, where one half was preserved in formalin for species identification the other half was dried and weighed for biomass estimation. Temperature, salinity and depth were also measured during the surveys at the same locations by a SEABIRD CTD sensor from the surface down to 500m or to the sea bottom (at shallower depths). However, the analyses below are limited to the uppermost 50 meters and applied as average temperature and salinity over 0–50 m for each station. In the Nordic Seas, the thermocline is generally at 20–40 m depth during summer [39]. The mackerel is typically found in this layer [10], and the trawling takes place there, thereby the stomach sampling. Instead of limiting the environmental variables to this layer (~40m) it was decided to use 0-50m. The reasons are: (1) mackerel can be expected to feed near and below the thermocline to some degree, especially within the Atlantic waters where the temperature below the thermocline is >7°C (SE, SW and W; Fig 1); (2) The mackerel can be expected to feed and occupy the whole well-mixed water column above the thermocline so using average temperature and salinity sounds logical; (3) The salinity is relatively unconnected to the thermocline so the average value over 50m represents mainly the type of water masses (Atlantic, Arctic, mixture or coastal).

### Dietary analysis

In this study, different indices previously used in dietary studies were applied to the stomach content data to address the different objectives by following Hyslop [40]. Numerical (%N) and gravimetric (%W) composition were calculated together with the frequency of occurrence (FOi), for all stomachs. To estimate the feeding activity of mackerel the Vacuity Index (Vi) was calculated;

$$V_{i}=\frac{E_{s}}{T_{s}}\times 100$$

where *E*_*s*,_ is the number of empty stomachs and *T*_*s*_ the total number of stomachs. The Prey-Specific Index of Relative Importance (*PSIRI*) was used to quantify the importance of each prey category in the diet [41]. *PSIRI* is ideal for comparisons between predators and prey because its values are not dependent upon the taxonomic level and act as a balanced treatment of the relative measures of prey quantity. *PSIRI* was calculated as:

$$PSIRI=(FO_{i}\times (PW_{i}+PN_{i}))/2$$

were the prey-specific abundance *PW*_*i*_ (weight) and *PN*_*i*_ (number) w needed for this calculation: *PW_i_* = ∑*W_i_*/∑*SW_i_*; *PNi* = ∑*N_i_*/∑*SN_i_* were *W*_*i*_ is the weight of prey *i* and *N*_*i*_ is the number of prey *i*. *SW*_*i*_ and *SN*_*i*_ are the total stomach content in weight and number, respectively, of individual predators with prey *i* in their stomachs. Feeding strategy was assessed graphically with a two-dimensional representation of prey-specific abundance (*P*_*i*_) and *FO*_*i*_ of the various preys [42]. The prey-specific abundance of prey *i (P*_*i*_*)* was defined as the percentage a prey taxon comprises of all prey items in only those predators in which the actual prey occurs. For this study, we used weight to describe *P*_*i*_, or in mathematical terms:

$$P_{i}=(\sum SW_{i}/\sum PW_{i})\times 100$$

### Statistical analysis

For statistical testing of diet contribution, fourth-root gravimetric data were assessed, using permutational multivariate analysis of variance (PERMANOVA; [43]) using a Bray-Curtis similarity index or a Kruskal Wallis followed by Dunn´s post hoc if significant. All statistical analyses were performed in PAST v3.20 [44] using a significance level of p < 0.05. Spatial differences in diet were also examined using the Bray-Curtis similarity index on fourth-root gravimetric data between areas for all years, and hierarchical agglomerative clustering was applied to the similarity matrix [45–47], were clustering was based on the group average cluster mode and visualised in a dendrogram using PAST v3.20 software.

The variation of stomach weight of mackerel was analysed using a generalised additive model (GAM) [48]. A GAM is simply a generalised linear model (GLM) with a linear predictor that is composed of a sum of smooth functions of the covariates, and thus particularly effective at modelling complex ecological relationships [49]. A GAM structure can be written as:

$$g(E(Y))=\beta_{0}+s_{1}(x_{1})+s_{2}(x_{2})+\cdots +s_{p}(x_{p})$$

Where *Y* is the dependent variable, *E*(*Y*) denotes the expected value and *g*(*Y*) signifies the link function that links the expected value to the explanatory variables *x*_*1*_,…,*x*_*p*_ (Table 1). The terms *s*_*1*_(*x*_*1*_), …,*s*_*p*_(*x*_*p*_) refer to smooth nonparametric functions.

**Table 1:** Variables and model selection.

**A)**								
**Variables**	**Description**							
Depth	Measured from sea surface to the ocean floor (meters)							
Sea surface salinity (SSS)	Average salinity from each station from 0–50 meters (parts "per mille"—ppt)							
Sea surface temperature (SST)	Average temperature from each station from 0–50 meters (°C)							
Zooplankton biomass	Dry weight from WP2 hauls at each station (mg/m3)							
Fulton's K	Fulton's condition factor (K = 100(W/L3)							
Latitude and longitude	Geographical marker of stations							
Total catch	Log transformed total mackerel catch (ton) per station							
Week	week number during the survey							
Time of day	Day divided into four time periods with 6 hours in each (00:00–05:00, 06:00–11:00, 12:00–17:00, 18:00–23:00)							
Year	Data collected from 2011–2014							
Distance to shore	From isobath lines (≤100m, ≤200m, ≤500, >501m)							
**B)**								
**Model #**	**Response variable Explanatory variable**	**Factorial variables**	**AIC**	**ΔAIC**	**w**_**i**_	**Deviance explained**	**R2 adjusted**	**DF**
1	log(stomach weight) zooplankton + (longitude,latitude)+ Fulton´s K+ depth + SSS + SST + log(total catch)	time period+distance to shore+week+year	-1854	8.92	0.007	48.50%	0.44	153
2	log(stomach weight) zooplankton + (longitude,latitude)+ depth + SSS + SST + log(total catch)	time period+distance to shore	-1863	0	0.58	48.60%	0.44	150
3	log(stomach weight) zooplankton + (longitude,latitude) + SSS + SST + log(total catch)	time period	-1862	1	0.35	48.50%	0.44	148
4	log(stomach weight) zooplankton + (longitude,latitude)+ depth+ SSS + SST	time period	-1860	4.4	0.06	48.30%	0.42	147

A) List of explanatory variables considered in analyses of Northeast Atlantic mackerel stomach weight in Icelandic waters in 2011–2014 using generalised additive models (GAMs). B) GAMs selection table, the model marked in bold was found to be the best-fitted model based on Akaike Information Criterion (AIC) from the R package “MuMIn” (see Table 6).

To examine which of the variables were significant in explaining the stomach weight of mackerel, we used data collected from each trawl station containing mackerel. Here the stomach weight is considered to represent feeding success. In our data, most of the stomachs contained prey, which suggests that mackerel that can feed continuously are in better condition and thus have better feeding success [50,51]. The explanatory variables included in the modelling were; time of sampling (four time periods (00:00–05:00, 06:00–11:00, 12:00–17:00, 18:00–23:00), week number and year), location (station and distance to shore (0–200m, 201–500m, 501–1000m, >1001m), environmental variables (bottom depth, average temperature from 0–50m and average salinity from 0–50m) and biological variables (zooplankton dry weight biomass, Fulton’s K (K = W / L^3^ x 100) of individual mackerel, total catch of mackerel) (Table 1A). Only years from 2011–2014 (S1 Table) were used in the GAMs since no zooplankton biomass data was available in 2009 and 2010. Also, only stations with > 9 individual stomachs were selected for all years for more robust comparisons. Furthermore, small fish (< 25 cm) were not included in the analysis because they were not well represented for all years in the dataset (S1 Fig). Before modelling, data were checked for collinearity.

The GAMs were fitted to a Gaussian distribution with an identity link function in the R package “mgcv” v. 1.8–26 [49], using Restricted Maximum Likelihood (REML) as smoothing selection [52]. The best-fitting GAM was selected by computing models with every possible combination of the variables using the “MuMIn” package v. 1.42.1 [53], as well as using visual assessment of the residual—and smooth plots and Akaike Information Criterion (AIC), delta (Δ) AIC and Akaike weight (*w*_*i*_) [54]. Variables were excluded if their inclusion did not belong within the 95% confidence set of the model (Table 1B).

## Results

### Diet composition and feeding strategy

The vacuity index (*VI*) was between 2–22% (average *VI* = 8.3%), with no statistical difference between years (Kruskal- Wallis; H = 5, p = 0.42) and overall, 92% of the mackerel stomachs contained food, meaning that almost all stomachs contained prey items to a varying degree. A total of 42 prey species were identified and are listed in Table 2. For further analysis, the prey species found in the stomachs were grouped into the ten following taxon groups; molluscs, copepods, amphipods, euphausiids, large crustaceans, small crustaceans, fish, appendicularians, chaetognaths and ova. All unidentified prey matter and parasites found in the stomachs were excluded from further analysis.

**Table 2:** Prey species observed in the stomach content of Northeast Atlantic mackerel in Icelandic waters in 2009–2014.

Group	* *	PWi%	PNi%	FOi%	PSIRI%
**Molluscs**		3.80%	1.20%	17.90%	0.40%
	*Planktomya* spp.				
	*Limacina helicina*				
	*Limacina retroversa*				
	*Prosobranchia* spp.				
	*Todarodes sagittatus*				
**Copepods**		72.90%	97.30%	81.30%	69.20%
	*Calanus finmarchicus *				
	*Calanus hyperborus*				
	*Acartia clausi*				
	*Temora longicornis*				
	*Centropages hamatus*				
	*Metridia longa *				
	*Oithona similis *				
	*Microcalanus* spp.				
	*Pseudocalanus* spp.				
	*Euchaeta spp*.				
	*Caligidae* spp.				
**Amphipods**		15.60%	2.20%	35.40%	3.10%
	*Themisto abyssorum*				
	*Hyperia medusarum*				
	*Gammaridae spp*.				
**Euphausiids**		18.60%	1.30%	40.10%	4.00%
	*Thysanoessa inermis *				
	*Thysanoessa longicaudata*				
	*Meganyctiphanes norvegica*				
**Large crustaceans**		42.10%	9.80%	21.20%	5.50%
	*Leucon (Leucon) nasica*				
	*Carcinus maenas *				
	*Hymenodora glacialis*				
	*Eusergestes arcticus*				
**Small crustaceans**		16.10%	4.40%	7.60%	0.80%
	*Balanidae* spp.				
	*Ostracoda* spp.				
	*Podon* spp.				
	*Evadne* spp.				
**Fish**		26.70%	0.30%	7.80%	1.10%
	*Ammodytes* spp.				
	*Clupea harengus*				
	*Gadus morhua*				
	*Melanogrammus aeglefinus *				
	*Micromesistius poutassou*				
	*Merlangius merlangus*				
	*Maulisia mauli*				
	*Mallotus villosus*				
	*Anarhichas minor*				
**Appendicularia**		16.30%	32.00%	1.40%	0.30%
	*Oikopleura* spp.				
**Chaetognaths**		16.20%	0.80%	1.00%	0.10%
	*Sagitta spp*.				
**Ova**		0.20%	1.20%	13.60%	0.10%
** **	*Actinopteri* spp.				

Observed prey and categorisation across species, showing prey-specific weight (*PW*_*i*_%) and number (*PN*_*i*_%), Frequency of Occurrence (*FO*_*i*_%) and Prey-Specific Index of Relative Importance (*PSIRI*%) of all years combined.

The overall prey specific index of relative importance (*PSIRI*) showed that the most common prey group that occurred in all mackerel stomachs for all the years was the copepod group (Fig 2), constituting a *PSIRI* between 48.7% to 86.6% in the years studied. Other important prey groups varied more among years in *PSIRI* with euphausiids ranging from 1.4–5.8%, large crustaceans 0.01–14.7%, amphipods 1.1–6.2% and fish 0.5–2.0%. The remaining prey groups, which had lower *PSIRI* values, were thus considered of lesser importance.

**Fig 3:**
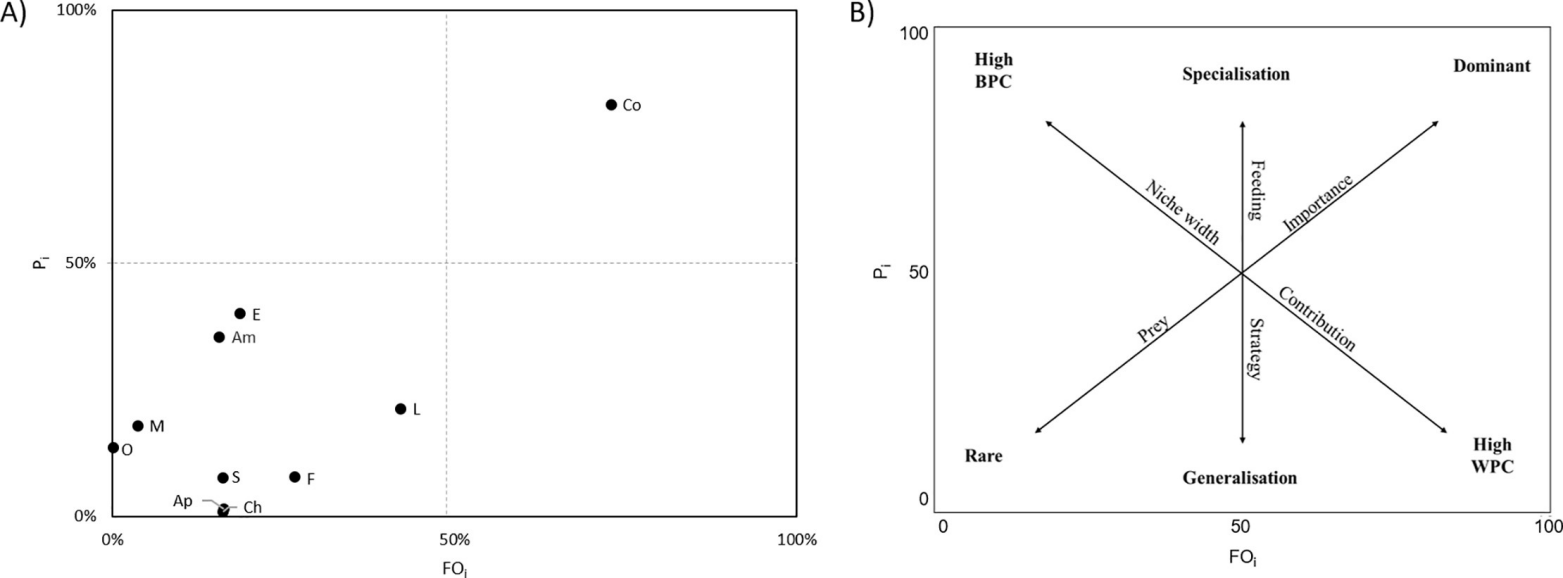
Graphical representation of feeding strategy from the stomach composition of Northeast Atlantic mackerel in Icelandic waters in 2019–2014. A) Feeding strategy shown by plotting frequency of occurrence (*FOi*%) and prey-specific abundance (*Pi*%) of prey in diet of fish collected where the prey groups are: M = molluscs; Co = copepods; Am = amphipods; E = euphausiids; L = large crustaceans; S = small crustaceans; F = fish; Ap = appendicularians; Ch = chaetognaths; O = ova. B) Explanatory diagram for interpretation of feeding strategy, prey importance and niche width contribution for mackerel (adapted from Amundsen et al. [42]); BPC, between-phenotype component; WPC, within-phenotype component.

To assess the feeding strategy of mackerel, the prey-specific abundance (*P*_*i*_) was plotted against the frequency of occurrence (*FO*_*i*_) (Fig 3A), which was done for the whole surveyed area in Icelandic waters pooling all years. Almost all prey groups were located to the lower left of the diagram, i.e. a region of low prey importance (Fig 3B). Copepods was the dominated prey group and its location at the upper right corner of the diagram (Fig 3A) signifies its importance and specialisation by the mackerel.

Results from the two-way PERMANOVA indicated a statistical difference in gravimetric weight of prey among areas and years (*Pseudo* F_4,5_ = 5.3, p<0.001; *Pseudo* F_4,5_ = 13.8, p<0.001). It also revealed that most prey groups varied among years and areas (Tables 3 and 4). Exceptions to this pattern include euphausiids among years and molluscs, large crustaceans, and chaetognaths among areas.

**Table 3:** Summary of two-way PERMANOVA for the analysis of differences between areas and years.

Source	*df*	*SS*	*MS*	*Pseudo-F*	*P*
*Molluscs*					
Area	4	1.26	0.32	1.15	>0.1
Year	5	6.68	1.34	5.00	**<0.001**
Residual	363	99.00	0.27		
*Copepods*					
Area	4	1.12	0.28	3.40	**<0.001**
Year	5	1.53	0.31	3.70	**<0.001**
Residual	363	30.00	0.08		
*Amphipods*					
Area	4	4.82	1.20	4.37	**<0.001**
Year	5	3.76	0.75	2.74	**<0.01**
Residual	363	99.91	0.28		
*Euphausiids*					
Area	4	2.96	0.74	3.00	**<0.01**
Year	5	1.39	0.28	1.10	>0.1
Residual	363	92.68	0.26		
*Large crustaceans*					
Area	4	0.74	0.18	0.95	>0.1
Year	5	38.86	7.77	40.17	**<0.001**
Residual	363	70.23	0.19		
*Small crustaceans*					
Area	4	2.08	0.52	2.60	**0.01**
Year	5	4.64	0.93	4.63	**<0.001**
Residual	363	72.70	0.20		
*Fish*					
Area	4	2.55	0.64	9.08	**<0.001**
Year	5	0.54	0.11	1.54	>0.1
Residual	363	25.48	0.07		
*Appendicularians*					
Area	4	0.84	0.21	4.51	**0.001**
Year	5	0.60	0.12	2.56	**<0.01**
Residual	363	17.00	0.05		
*Chaetognaths*					
Area	4	0.23	0.06	1.13	>0.1
Year	5	0.93	0.19	3.61	**0.001**
Residual	363	18.64	0.05		
*Ova*					
Area	4	1.71	0.43	1.51	0.1
Year	5	13.72	2.74	9.69	**<0.001**
Residual	363	102.80	0.28		

Based on Bray–Curtis dissimilarities of the fourth-root gravimetric weight of prey groups of mackerel in 2009–2014 in Icelandic waters. Significant results are shown in bold.

**Table 4:** Pairwise comparisons from the results of the two-way PERMANOVA (Table 3).

**A)**					*Large Crustaceans*	*Small Crustaceans*		*Appen-dicularians*	*Chaet-ognaths*	
	*Molluscs*	*Copepods*	*Amphipods*	*Euphausiids*			*Fish*			*Ova*
*2009–2010*	**0.001**	**<0.001**	**<0.01**	>0.5	**<0.001**	>0.1	**<0.05**	0.5	**<0.01**	>0.1
*2009–2011*	**<0.001**	**<0.05**	>0.1	>0.5	**<0.001**	**0.001**	>0.5	**0.01**	>0.1	>0.05
*2009–2012*	**<0.001**	>0.5	>0.1	>0.05	**<0.001**	>0.5	>0.1	**0.01**	>0.1	>0.1
*2009–2013*	**<0.05**	**<0.05**	>0.5	>0.5	**0.01**	>0.1	**0.001**	1	**0.05**	**<0.001**
*2009–2014*	**0.001**	>0.5	>0.1	>0.5	**<0.05**	>0.1	**<0.001**	**0.01**	>0.1	**<0.001**
*2010–2011*	>0.1	>0.05	>0.1	>0.5	**0.01**	**<0.001**	>0.1	>0.1	1	**0.01**
*2010–2012*	**0.02**	**<0.01**	**<0.001**	>0.1	>0.05	>0.1	>0.5	>0.1	**<0.001**	>0.05
*2010–2013*	>0.1	**<0.001**	**<0.01**	>0.5	**<0.001**	**<0.05**	>0.05	>0.5	**<0.01**	**<0.001**
*2010–2014*	>0.1	**0.001**	>0.05	>0.1	**<0.001**	**0.01**	>0.05	>0.1	>0.1	**<0.001**
*2011–2012*	>0.1	>0.05	**<0.05**	>0.1	>0.1	**0.001**	>0.1	1	**<0.001**	>0.1
*2011–2013*	>0.1	**0.001**	>0.1	>0.5	**<0.001**	**<0.05**	**<0.01**	**0.01**	**<0.01**	**<0.001**
*2011–2014*	>0.5	**<0.05**	>0.5	>0.1	**<0.001**	**<0.05**	**<0.01**	>0.5	>0.1	**0.01**
*2012–2013*	**0.01**	**0.01**	>0.1	>0.05	**<0.001**	>0.1	**<0.05**	>0.05	>0.5	**<0.001**
*2012–2014*	>0.05	>0.05	**<0.5**	**<0.05**	**<0.001**	>0.1	**<0.05**	>0.5	**<0.05**	**<0.01**
*2013–2014*	0.5	>0.1	>0.1	>0.5	>0.5	>0.5	>0.5	>0.05	**0.01**	>0.05
**B)**					*Large Crustaceans*	*Small Crustaceans*		*Appen-dicularians*	*Chaet-ognaths*	
	*Molluscs*	*Copepods*	*Amphipods*	*Euphausiids*			*Fish*			*Ova*
*W-SW*	>0.5	>0.1	**<0.001**	>0.5	>0.1	>0.1	>0.1	**0.01**	>0.1	>0.1
*W-SE*	>0.5	>0.1	**0.01**	>0.1	>0.5	**0.01**	>0.5	**<0.01**	>0.5	>0.05
*W-E*	>0.1	**0.01**	**0.001**	>0.05	>0.5	>0.1	>0.5	**0.01**	>0.1	>0.5
*W-N*	>0.05	**<0.001**	>0.1	**<0.01**	>0.1	>0.1	**0.001**	>0.5	>0.5	>0.5
*SW-SE*	>0.5	>0.5	>0.05	>0.1	>0.1	>0.1	>0.05	1	>0.5	>0.5
*SW-E*	>0.1	>0.1	**<0.05**	>0.05	>0.1	>0.1	>0.1	>0.1	**0.05**	>0.1
*SW-N*	>0.1	**0.01**	**<0.01**	**0.01**	>0.5	>0.05	**<0.001**	**<0.05**	>0.1	>0.1
*SE-E*	>0.5	>0.1	>0.1	**0.05**	>0.5	**<0.05**	>0.1	>0.1	>0.1	**<0.05**
*SE-N*	>0.1	>0.05	>0.1	**0.01**	>0.1	**0.001**	**<0.05**	**<0.05**	>0.5	**<0.05**
*E-N*	>0.1	**0.01**	>0.1	>0.1	>0.1	>0.1	**<0.001**	>0.1	>0.1	>0.5

Based on Bray–Curtis dissimilarities of fourth-root transformed values of gravimetric weight of prey between years (A) and between areas (B) for all years combined. Significant results are shown in bold.

The difference between the three length groups of mackerel in the relative measure of total stomach content, indicated by the gravimetric index, was only significant in 2011 (H = 52.1, p<0.001) and in 2012 (H = 10.9, p<0.005). Dunn´s post hoc test showed a difference between all length groups in 2011 (S-M, p<0.001; S-L, p<0.001; M-L, p<0.005) while in 2012 there was a difference between the largest mackerel and the two other length groups (L-S, p<0.005; L-M, p<0.01). When analysing differences in prey composition among length groups combined over all the years, there was only difference with the fish prey group (H = 13,1, p<0.005), where a Dunn´s post hoc revealed that larger mackerel preyed more on fish than smaller mackerel did (S-M, p<0.05; S-L, p<0.001).

### Diet variation between areas

**Fig 4:**
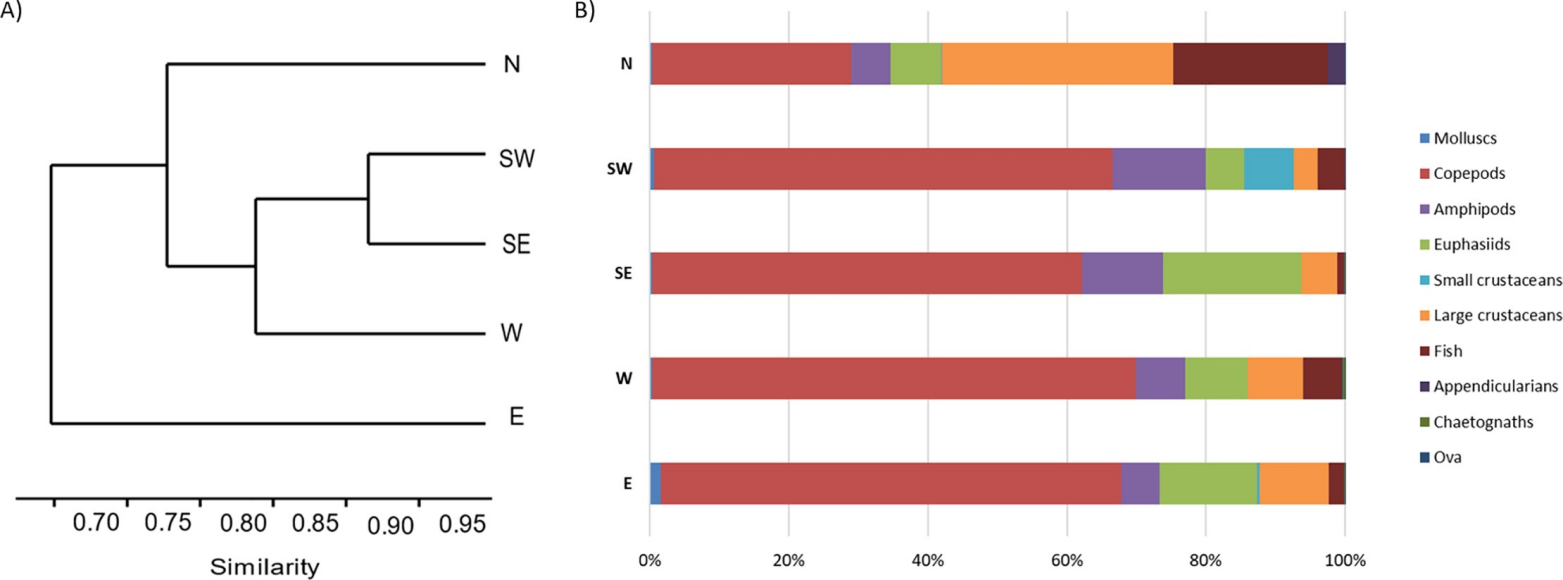
Diet variation between areas. A) Dendrogram for hierarchical clustering of the prey composition of mackerel according to sampling locations (Fig 1) using Bray–Curtis similarities calculated on fourth-root transformed values of the gravimetric weight of prey. B) Composition of mackerel diet by area, based on the gravimetric weight of prey (W%).

Results from the one-way PERMANOVA analysis of prey composition in the stomachs showed that there was some diet variation between areas within the years (Table 5 and Supplementary S2 and S3 Tables). Hierarchical clustering of the five areas based on the diet variation resulted in three groups that were clearly separated but still showed a high percentage of similarity (Fig 4A). One of the groups consisted of west, southwest and southeast areas with 85–92% similarity, the second group (north) revealed slightly lower similarity (78%) and the lowest being east (70%). These similarities were also recognised by a graphical representation of the diet composition in each area (Fig 4B).

**Table 5:** One-way PERMANOVA of species composition from stomachs between areas within years.

Prey groups	2009	2010	2011	2012	2013	2014
*Molluscs*	>0.5	>0.1	**<0.001**	**<0.05**	>0.1	>0.1
*Copepods*	>0.5	>0.05	**<0.01**	**0.01**	>0.1	>0.5
*Amphipods*	**0.05**	>0.1	>0.05	**0.01**	>0.05	>0.5
*Euphausiids*	**0.01**	>0.1	>0.1	>0.5	**<0.01**	>0.1
*Large crustaceans*	>0.1	**<0.01**	>0.05	>0.5	>0.5	>0.5
*Small crustaceans*	0.1	>0.05	>0.1	>0.1	>0.5	**<0.05**
*Fish*	>0.1	**<0.05**	**0.01**	>0.1	>0.1	0.5
*Appendicularians*	*NA*	**<0.05**	>0.1	>0.05	*NA*	>0.05
*Chaetognaths*	>0.5	*NA*	*NA*	>0.1	>0.5	>0.1
*Ova*	>0.1	>0.1	>0.05	>0.1	>0.1	>0.5

Based on Bray–Curtis dissimilarities of fourth-root gravimetric weight of prey. Significant results are shown in bold.

### Variation in stomach weight

**Fig 5:**
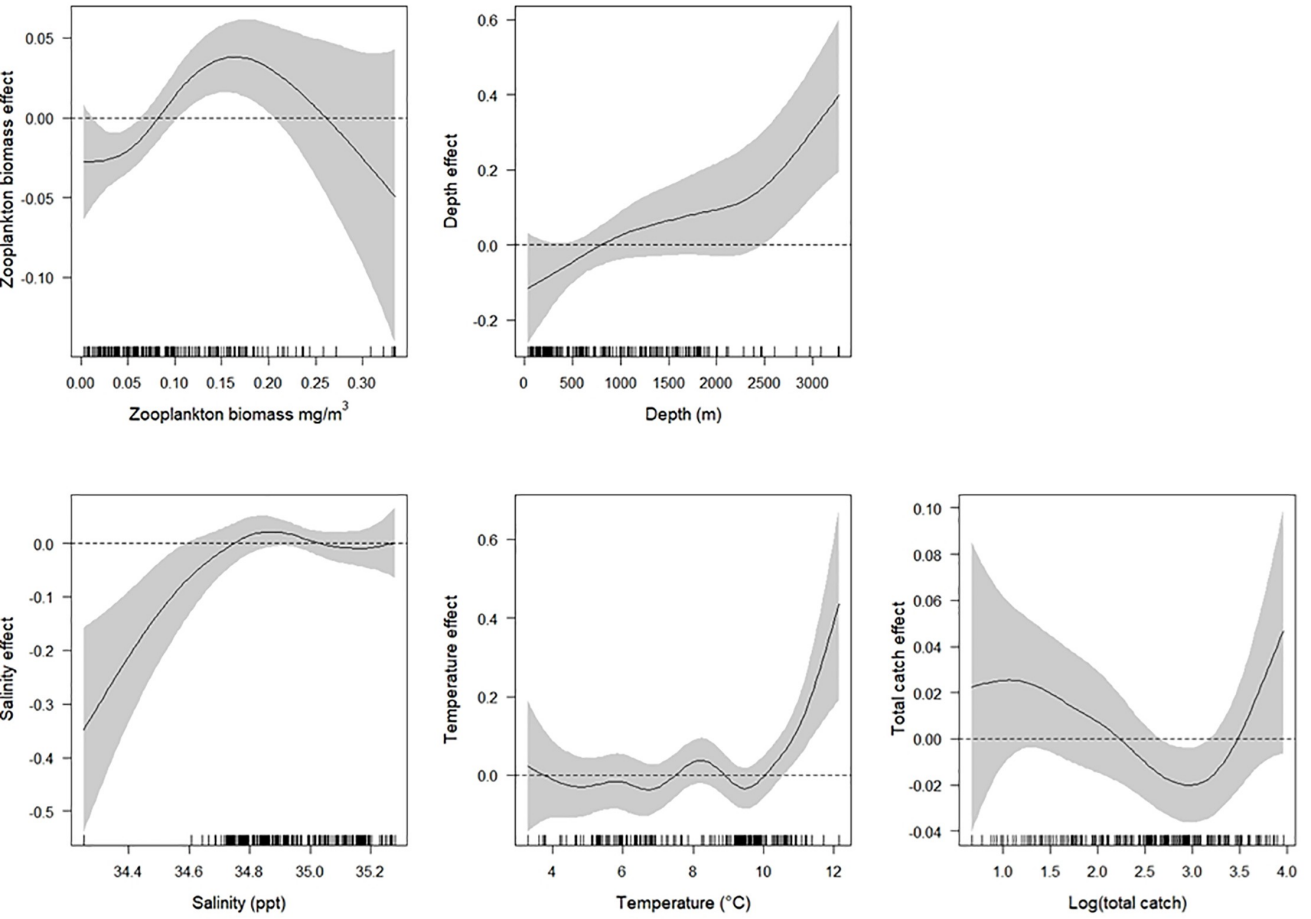
Generalised additive model (GAM). Results from the GAM of the effects of different explanatory variables on Northeast Atlantic mackerel stomach weights in the summers of 2011–2014 in Icelandic waters, where the solid lines are smoother estimates of the covariates according to the model. The shaded grey area represents the 95% confidence interval of the smoothers, and vertical dashes at the bottom of the plots show the distribution of data points entering the model.

The results from the GAMs, show that the stomach weight of mackerel was primarily affected by zooplankton biomass, temperature, salinity, depth as well as spatial and temporal variables, in this order, which together explains over 48% of the deviance. All predictor variables, except total catch per station, contributed to the overall model by having a smoothing term significantly different from zero (Table 6). Increase in zooplankton biomass had a positive effect on stomach weight, while a negative trend, with a wide confidence interval, was detected in the higher end of the zooplankton biomass based on few samples (Fig 5). Depth had an overall positive effect on the stomach weight of mackerel, whereas the positive relationship between salinity and stomach weight was largely driven by a single station with low values for both variables. Temperature had a more mixed effect but became positive when it exceeded 9°C. The total catch had both a negative and positive effect on stomach weight. Regarding the factorial variables, some significant effects were revealed, both regarding the distance to the shore (depth profiles) and time of day (time period).

**Table 6:** Summary statistics from the general additive model (GAM).

**A) Parametric coefficients**	**Estimate**	**Std. error**	**t-value**	**p-value**
Intercept	1.00986	0.05624	17.955	**<0.001**
Distance to shore2 (depth 200–500m)	-0.07544	0.0306	-2.465	**<0.05**
Distance to shore3 (depth 501-1000m)	-0.14472	0.06793	-2.13	**<0.05**
Distance to shore4 (depth > 1000m)	-0.20338	0.09313	-2.184	**<0.05**
Time period 2 (06:00–11:00h)	-0.02611	0.01839	-1.42	>0.1
Time period 3 (12:00–17:00h)	-0.02054	0.01763	-1.165	>0.1
Time period 4 (18:00–23:00h)	-0.06468	0.01583	-4.086	**<0.001**
**B) Smooth terms**	**Est. DF**		**F**	**p-value**
s(zooplankton biomass)	3.174		4.376	**<0.01**
s(bottom depth)	3.227		4.818	**<0.01**
s(salinity)	3.594		3.803	**<0.01**
s(temperature)	116.947		5.897	**<0.001**
s(longitude, latitude)	6.48		3.286	**<0.01**
s(total catch)	3.183		2.85	>0.05

Showing the parametric coefficients of factorial variables (A) together with the approximate significance of smooth terms used in the model (B). Significant values are in bold.

## Discussion

### The variation in prey composition

The results showed that mackerel’s main food source while feeding in Icelandic waters, were calanoid copepods, which constituted (on average) >60% of the total content weight of the stomachs. Over 70% of the copepods by weight consisted of *C*. *finmarchicus*. This finding is in accordance with studies done on the zooplankton community in Icelandic waters, which have identified *C*. *finmarchicus* to be the most abundant calanoid species (50–80%) [55,56]. Other diet studies of mackerel from other areas in the Northeast Atlantic showed similar results, where *C*. *finmarchicus* was found to be the most dominant copepod species in mackerel stomachs [16,20,26,57,58]. Smaller copepods, such as *T*. *longicornis* and *Oithona* spp., were also found in large amounts in mackerel stomachs during their summer feeding, especially from those fish caught close to and on the shelf area to the south and southeast. This coincides with similar diet studies of mackerel from the Norwegian Sea [57] and in the Gulf of St. Lawrence [59,60] and characterises their more coastal distribution [7,61–63]. Mackerel are mainly planktivores, using both filter and particulate methods of feeding when faced with different prey assemblages in order to maximise their net intake [18]. Studies have shown that mackerel may feed on zooplankton by primarily filter feeding and then shift to particulate feeding when prey size and the size distribution of available prey changes [18,19], which probably allows them to make more extensive use of the available prey. Therefore, the feeding behaviour of mackerel can be size-selective and is probably based upon the efficiency of retention of different-sized particles by the gill rakers.

This study revealed that euphausiids, amphipods and large crustaceans play an essential role as food for mackerel. Of the four euphausiid species found in Icelandic waters, *Thysanoessa inermis* occurs all over the Icelandic shelf, *T*. *longicaudata* is most abundant in offshore waters, *T*. *raschi* is mainly confined to fjords and bays and *Meganyctiphanes norvegica* is most common near the shelf edge off the south and west coasts [64,65]. The euphausiid species identified in the analysed stomachs was, to a large extent *T*. *inermis* and to some extent *M*. *norvegica*. *Themisto abyssorum* was the most abundant amphipod species in the stomachs, which concurs very well with zooplankton studies finding *T*. *abyssorum* to be the most common amphipod species in Icelandic waters [66]. In 2010–2012, megalopa larvae of large crustaceans (mostly crab and shrimp), were found to a greater extent (second most important prey item (*PSIRI*) Fig 2) in the stomach content than other years. It is difficult to say why this prey group was represented in greater abundance in those three years, than in the other years. The only shrimp and crab species fished commercially, and thus monitored regularly, in Icelandic waters are the Northern shrimp (*Pandalus borealis*) that are found all around Iceland but more abundance in the north and east [67] and Norway lobster (*Nephrops norvegicus*) which is, however, limited to the south and west coast of Iceland [64]. We cannot tell with the data at hand, if and how much of these observed megalopa larvae belong to these species, and thereby if, for example, the mackerel predation could be impacting the Norway lobster recruitment success, which has been failing for many years [68]. However, this is of both ecological and economic interest and requires further investigation through, for example, genetic analysis of stomach content, to assess the potential impact.

Fish were the only prey group that varied among length groups, where large mackerel (≥39cm) tended to consume more fish than smaller mackerel. Experiments on mackerel foraged on fish larvae revealed that adult mackerel actively shifted preference towards larger prey to achieve a higher rate of energy intake, and thus preyed more heavily on large larvae than the smaller mackerel [19,24]. This strategy could be more profitable for larger fish, who could endure the extra energy it takes, to actively feed on more mobile prey. Otherwise, there was not much difference in diet composition between the length groups, which could be because most mackerel caught in Icelandic waters during the summer are relatively large (>33cm) (S1 Fig). Smaller and younger mackerel occasionally observed in Icelandic waters (<25 cm [69]), were not part of this study.

The results of prey composition in mackerel stomachs between areas revealed some subtle differences (Fig 4) and these differences reflect the zooplankton distribution pattern around Iceland to some degree. Past studies have shown that the mean annual zooplankton biomass in the surface layers are more than two times higher in the warm Atlantic water south of Iceland than in the subarctic waters to the north and that species composition varies greatly among areas as well [7,38]. In the uppermost water mass around Iceland *C*. *finmarchicus* is the most dominant zooplankton species, followed by *Oithona* spp. [38,63]. Other species like *T*. *longicornis*, *Centropages hamatus*, *Acartia* spp., *Podon* spp., cirripede larvae, bivalves and polychaetes dominate the biomass to the southeast, south and southwest [7,38,63,64]. To the north and northeast of Iceland, there seem to be relatively high zooplankton biomass of *C*. *hyperboreus*, *C*. *glacialis*, *Metridia longa*, *Pseudocalanus* spp., euphausiids and appendicularia (*Oikopleura* spp.) [7,38,63–65]. Although *C*. *finmarchicus* was the dominant copepod species found in mackerel stomachs for all areas, smaller copepod species such as *T*. *longicornis* and *Arcatia clausi* were especially abundant in samples from the coastal areas to the southwest and southeast. As were small crustaceans (i.e. cirripede larvae and *Podon* spp). In the northern area, the diet was also dominated by large crustaceans and fish, where euphausiids were more commonly found in stomachs from the east and southeast.

### Feeding strategy of mackerel

The vacuity index (VI) (or empty stomachs’ ratio), is an inverse indicator of feeding intensity which varies according to variations in the abundance of fish as well as seasonal changes in water temperature and food items available [70]. The low value of the vacuity index (VI) for all years indicate that mackerel feed continuously while in Icelandic waters during the summer. Mackerel is a visual predator, and experiments have shown that mackerel have a greater feeding activity during the day than at night. At the time they are foraging in Icelandic waters, there is daylight almost for 24 hours, which allows them a more continuous feeding time frame [19,59]. Our findings from the GAM modelling supports this where the time of the day had only small impacts on the variation in the stomach weight. Only time period 4 (from 18:00–23:00 hours) was found significant, with a tendency for less stomach weight indicating, surprisingly, a less feeding in the afternoon (Table 6). Study of mackerel feeding behaviour from the Irminger Sea by Jansen et al. (2019) [50] during the summer, found that mackerel had some diel aspects in their feeding dynamics, where they consumed larger zooplankton prey during dusk hours, even though copepods were still numerous in the surface layers. Our data on stomach composition did not allow for analyses of the diurnal differences.

Our general findings from the GAM indicated that the weight of mackerel stomachs were affected by several explanatory variables. The positive impact of the sea temperature was not surprising since mackerel prefer higher temperatures. In the same way, zooplankton biomass had a positive effect on the stomach weight up to a certain degree, where it started to have a negative effect according to the model. The positive effect seems like logical, where higher density of zooplankton causes higher feeding success and thereby greater stomach weight. This negative effect was, however, caused by few samples, had wide confidence interval, and is therefore considered to have little significance. There are some shortcomings in our approach that should be mentioned regarding the zooplankton biomass. It was obtained from WP2 plankton nets, and thus not fully representative of the zooplankton community, since larger zooplankton species, like euphausiids, tend to evade capture [71]. The results from the GAM are interesting enough for further exploration regarding the feeding of mackerel in Icelandic waters concerning shifts in time of day as well as the waters they occupy (coastal, shelf or oceanic).

Analyses of the feeding strategy of mackerel showed that it is a specialised predator during the summer feeding, that relies heavily upon copepods as its main prey while eating other prey groups to a varying degree when encountering them. Since mackerel has the option to both filter and particulate feed, it can switch between methods readily, in order to utilise the searched area (Macy et al. 1998; Darbyson et al. 2003). Feeding experiments have shown that when mackerel were introduced to high concentrations of large copepods, they switched from particulate feeding to filter feeding (Macy et al. 1998). This was also true when offered intermediate and smaller prey items at relatively high concentrations, and when the concentration of smaller size zooplankton is low, mackerel shift back to particulate feeding [19]. The shift in feeding method can be explained by the energy cost of filter feeding [72] as well as a lower energy density of *C*. *finmarchicus* [73]. Thus, mackerel can shift to larger and more energy-rich food sources when available. This has also been observed in nature, where mackerel switched to particulate feeding of large zooplankton even though copepods were available [50]. These types of behaviour responses are also thought to be common within mackerel schools, because differences in the size and abundance of prey at the front and rear end of schools may vary [18]. Studies have also shown that mackerel tend to slow their swimming speed when encountering highly concentrated patches of zooplankton [14,18], probably as they do not need to “chase” their prey. Higher swimming speeds are then associated with larger prey and reflect a higher energy intake [18,19]. Thus, mackerel increase their swimming speed as zooplankton abundance decreases, and thereby tend to have high clearance rates of a variety of prey sizes within the plankton community, which then again can affect the density of less abundant prey such as fish larvae. In summary, the literature implies that mackerel is an opportunistic predator, meaning that is generally not selective in its feeding but more or less feeds on the biomass available. Our results indicate that mackerel is a specialised predator on copepods, although this can also be interpreted as support to the opportunistic feeding strategy since the most abundant biomass of zooplankton prey in Icelandic waters are calanoid copepods.

### Studies limitations

Evacuation studies have shown that adult mackerel can clear their stomachs within 28 hours at 17°C but longer evacuation time is needed at lower temperatures [74,75]. Furthermore, mackerel evacuate stomach content at a continuous rate, and smaller prey items are evacuated faster than large ones [75]. Therefore, visual analysis of stomach contents alone can be biased, because often the contents are so digested that there is only “soup”, or bits and pieces left visible. This makes it hard to quantify and identify the prey down to species level.

Consequently, a more comprehensive and alternative analysis of a fish´s diet can be informative and relevant, not only in strengthening and widening the results, but also to discover if there are prey species that were not detectable by visual analysis alone. These methods could be in the form of stable isotope analysis of different fish tissues [76,77], fatty acid profiling [78,79] or genetic analysis of stomach content [80]. In this study, conventional methods were used to evaluate the feeding strategy of the Northeast Atlantic mackerel from stomach content analyses, which showed that mackerel is a specialised feeder upon copepods while in Icelandic waters. These findings correspond very well with similar studies on mackerel diet in the Northeast Atlantic during the summer. However, applying different methods might give a different perception and more holistic information on the temporal and spatial variability on the prey field of Northeast Atlantic mackerel across its distribution area.

### Ecological considerations

Mackerel, being a ferocious feeder and capable of sustaining high clearance rates of prey items in their vicinity, can most likely have significant impacts on the marine ecosystem around Iceland and elsewhere [20,73]. Even though there are presently no documented cases of direct ecological impacts of mackerel in Icelandic waters, it seems logical to suggest that the relative recent massive influx of mackerel into Icelandic waters during their feeding migration, has altered the food web structure by top-down forcing, adding pressure on other planktivorous fish species as well. For example, the Norwegian spring spawning herring undertakes an extensive feeding migration in the early summer, from its spawning grounds along the Norwegian coast into the Norwegian Sea and to the waters east and north of Iceland, feeding mainly on overwintering *C*. *finmarchicus* [26,81]. As the summer progresses, herring tend to shift their diet to include more euphausiids and amphipods, although copepods are still an essential part of their diet [20,56]. Mackerel, on the other hand, arrives into Icelandic waters later and are mainly feeding on the first generation of *C*. *finmarchicus*, which is the main proportion of their diet throughout the summer [16,20,57]. Therefore, not only are mackerel competing with other species, (e.g. herring) for food, they have the potential to overgraze on the zooplankton community [73]. This could lead to a decline in zooplankton abundance, especially of *C*. *finmarchicus*, which in turn can have detrimental effects on the food-web structure, as well as the survival of many marine fish, bird and whale species [29,30,82–84]. Studies have shown, that mackerel on several occasions seem to prefer fish larvae to zooplankton [24,27], and reports say that mackerel readily feed on juvenile herring, capelin, sand eel among others [27,57,73,85]. Mackerel could, therefore, impact the survival of small fish and larvae in Icelandic waters as well. On the other hand, the increase of mackerel has also shown some positive effects on top predators such as seals, whales and northern gannets [29,83,86,87]. The ecological consequences of mackerel entering into Icelandic waters during their summer feeding migration are unknown, but given that they are avid foragers on the zooplankton community, the potential impact is imminent and should be taken into consideration for future research.

## Supporting information

S1 Table Sample sizes from the years 2009–2014 used in this study. (DOCX)

S2 Table Results of the Permutational Analysis of Variance (PERMANOVA) based on Bray–Curtis dissimilarities of fourth‐root transformed values of gravimetric weight of the prey within each year, between the five areas. Significant values are marked in grey
(DOCX)

S3 Table Results of pairwise comparison of the PERMANOVA (S2 Table), based on Bray–Curtis dissimilarities of fourth‐root transformed gravimetric weight of prey between areas within each year. Only prey groups that were significant from the PERMANOVA are listed. Significant values are marked in grey
(DOCX)

S1 Fig The size distribution of mackerel caught in Icelandic waters from 2009–2014 during the summer. (TIF)
