# Supplementary material for: Diet and feeding strategy of Northeast Atlantic mackerel (Scombrus scomber) in Icelandic waters
Source: PLoS One. 2019 Dec 30;14(12):e0225552. doi: 10.1371/journal.pone.0225552 (PMC6937200; doi:10.1371/journal.pone.0225552)
Supplement: S1 Table — (DOCX) [file pone.0225552.s001.docx]

| **Year** | **Measured** | **Visual analysis** | **GAM** |
| --- | --- | --- | --- |
| 2009 | 3754 | 821 | N/A |
| 2010 | 3453 | 743 | N/A |
| 2011 | 2963 | 637 | 505 |
| 2012 | 2870 | 516 | 376 |
| 2013 | 2906 | 499 | 399 |
| 2014 | 2655 | 561 | 517 |
| **Total** | **18601** | **3777** | **1797** |
|  |  |  |  |
