# Supplementary material for: Diet and feeding strategy of Northeast Atlantic mackerel (Scombrus scomber) in Icelandic waters
Source: PLoS One. 2019 Dec 30;14(12):e0225552. doi: 10.1371/journal.pone.0225552 (PMC6937200; doi:10.1371/journal.pone.0225552)
Supplement: S2 Table — Significant values are marked in grey (DOCX) [file pone.0225552.s002.docx]

|  | ***Molluscs*** | | ***Copepods*** | | ***Amphipods*** | | ***Euphausiids*** | | ***Large Crustaceans*** | | ***Small Crustaceans*** | | ***Fish*** | | ***Appendicularians*** | | ***Chaetognaths*** | | ***Ova*** | |
| --- | --- | --- | --- | --- | --- | --- | --- | --- | --- | --- | --- | --- | --- | --- | --- | --- | --- | --- | --- | --- |
|  | **F** | **p** | **F** | **p** | **F** | **p** | **F** | **p** | **F** | **p** | **F** | **p** | **F** | **p** | **F** | **p** | **F** | **p** | **F** | **p** |
| **2009** | 0.5 | >0.5 | 0.7 | >0.5 | 2.2 | <0.05 | 3.1 | 0.01 | 1.0 | >0.1 | 1.9 | >0.05 | 0.9 | >0.1 | NA | NA | 0.5 | >0.5 | 0.9 | >0.1 |
| **2010** | 1.0 | >0.1 | 1.8 | >0.05 | 1.6 | >0.1 | 1.7 | >0.1 | 3.6 | <0.01 | 2.3 | >0.05 | 1.6 | <0.05 | 3.2 | <0.05 | NA | NA | 1.6 | >0.1 |
| **2011** | 5.7 | <0.001 | 3.1 | <0.01 | 2.0 | >0.05 | 1.0 | >0.1 | 2.2 | >0.05 | 1.2 | >0.1 | 2.4 | <0.05 | 1.1 | >0.1 | NA | NA | 2.4 | >0.05 |
| **2012** | 3.1 | <0.05 | 3.00 | <0.05 | 3.3 | <0.05 | 0.7 | >0.5 | 0.6 | >0.5 | 1.4 | >0.1 | 0.9 | >0.1 | 2.0 | >0.05 | 1.1 | >0.1 | 0.9 | >0.1 |
| **2013** | 1.7 | >0.1 | 1.2 | >0.1 | 2.0 | >0.05 | 4.0 | <0.01 | 0.3 | >0.5 | 0.6 | >0.5 | 1.4 | >0.1 | NA | NA | 0.7 | >0.5 | 1.4 | >0.1 |
| **2014** | 1.3 | >0.1 | 0.7 | >0.5 | 0.8 | >0.5 | 1.1 | >0.1 | 0.7 | >0.5 | 3.2 | <0.05 | 0.4 | >0.1 | 1.9 | >0.05 | 1.0 | >0.1 | 0.4 | >0.5 |
