# Supplementary material for: Diet and feeding strategy of Northeast Atlantic mackerel (Scombrus scomber) in Icelandic waters
Source: PLoS One. 2019 Dec 30;14(12):e0225552. doi: 10.1371/journal.pone.0225552 (PMC6937200; doi:10.1371/journal.pone.0225552)
Supplement: S3 Table — Only prey groups that were significant from the PERMANOVA are listed. Significant values are marked in grey (DOCX) [file pone.0225552.s003.docx]

|  | **2009** | | | | **2010** | | | | **2011** | | | | | | **2012** | | | | | | **2013** | | **2014** | |
| --- | --- | --- | --- | --- | --- | --- | --- | --- | --- | --- | --- | --- | --- | --- | --- | --- | --- | --- | --- | --- | --- | --- | --- | --- |
|  | ***Amphipods*** | | ***Euphausiids*** | | ***Large Crustaceans*** | | ***Fish*** | | ***Molluscs*** | | ***Copepods*** | | ***Fish*** | | ***Molluscs*** | | ***Copepods*** | | ***Amphipods*** | | ***Euphausiids*** | | ***Small Crustaceans*** | |
| **Area** | **F** | **p** | **F** | **p** | **F** | **p** | **F** | **p** | **F** | **p** | **F** | **p** | **F** | **p** | **F** | **p** | **F** | **p** | **F** | **p** | **F** | **p** | **F** | **p** |
| ***W-SW*** | 3.6 | >0.1 | 1.3 | >0.1 | 7.5 | <0.01 | 0.1 | >0.5 | 0.5 | >0.5 | 1.2 | >0.1 | 0.3 | >0.5 | 1.4 | >0.1 | 1.6 | >0.1 | 11.8 | <0.01 | 3.6 | <0.05 | 1.7 | >0.1 |
| ***W-SE*** | 7.6 | <0.05 | 5 | <0.01 | 2.5 | >0.05 | 0.1 | >0.5 | 1.3 | >0.1 | 9.8 | <0.01 | 0.1 | >0.5 | 0.1 | >0.5 | 0.01 | >0.5 | 12.8 | <0.01 | 3.9 | <0.05 | 0.02 | >0.5 |
| ***W-E*** | 4.2 | <0.05 | 0.4 | >0.5 | 8.3 | <0.001 | 0.2 | >0.5 | 27.3 | <0.001 | 11.3 | <0.001 | 7.1 | <0.01 | 5.6 | <0.05 | 0.9 | >0.1 | 4.7 | <0.05 | 9.8 | <0.001 | 0.4 | >0.5 |
| ***W-N*** | 2.3 | >0.05 | 3.6 | <0.05 | 2.4 | >0.1 | 8.1 | <0.05 | 3 | >0.05 | 3.8 | <0.01 | 1.8 | >0.1 | 10.6 | <0.01 | 4.9 | <0.01 | 4.4 | <0.05 | 31.7 | >0.05 | 5.8 | <0.05 |
| ***SW-SE*** | 0.2 | >0.5 | 5.9 | <0.01 | 2.9 | <0.05 | 0.01 | >0.5 | 0.2 | >0.5 | 1.4 | >0.1 | 0.2 | >0.5 | 0.5 | >0.1 | 0.9 | >0.5 | 0.1 | >0.5 | 0.9 | >0.1 | 1.6 | >0.1 |
| ***SW-E*** | 0.3 | >0.5 | 1 | >0.1 | 0.5 | >0.5 | 0.1 | >0.5 | 13.1 | <0.001 | 3.8 | >0.05 | 3.5 | >0.1 | 0.8 | >0.1 | 1.8 | >0.1 | 1.8 | >0.1 | 5.4 | <0.05 | 2.9 | >0.1 |
| ***SW-N*** | 0.7 | >0.5 | 0.3 | >0.5 | 2.1 | >0.05 | 5.8 | <0.05 | 0.6 | >0.5 | 3.2 | >0.05 | 2.7 | >0.1 | 2.4 | >0.1 | 1 | >0.1 | 1.6 | >0.1 | 6.9 | >0.1 | 16.5 | <0.01 |
| ***SE-E*** | 1.3 | >0.1 | 3.8 | <0.05 | 1.7 | >0.1 | 0.04 | >0.5 | 8.2 | <0.01 | 0.9 | >0.1 | 5.6 | <0.05 | 2.6 | >0.1 | 0.5 | >0.5 | 1.8 | >0.1 | 1.6 | >0.1 | 0.4 | >0.5 |
| ***SE-N*** | 2.3 | >0.1 | 9.9 | <0.01 | 0.1 | >0.5 | 5.1 | <0.05 | 0.2 | >0.5 | 0.7 | >0.5 | 1.7 | >0.1 | 5.3 | <0.05 | 2.8 | <0.05 | 1.7 | >0.1 | 2.4 | >0.1 | 6.5 | <0.05 |
| ***E-N*** | 1.9 | >0.1 | 4.2 | <0.01 | 0.9 | >0.1 | 7.6 | <0.01 | 7.1 | <0.01 | 0.01 | >0.5 | 17.7 | <0.001 | 0.8 | >0.1 | 5.6 | <0.01 | 0.03 | >0.5 | 0.8 | >0.5 | 4 | >0.05 |
