# Supplementary figures and images for: Diet and feeding strategy of Northeast Atlantic mackerel (Scombrus scomber) in Icelandic waters
Source: PLoS One. 2019 Dec 30;14(12):e0225552. doi: 10.1371/journal.pone.0225552 (PMC6937200; doi:10.1371/journal.pone.0225552)

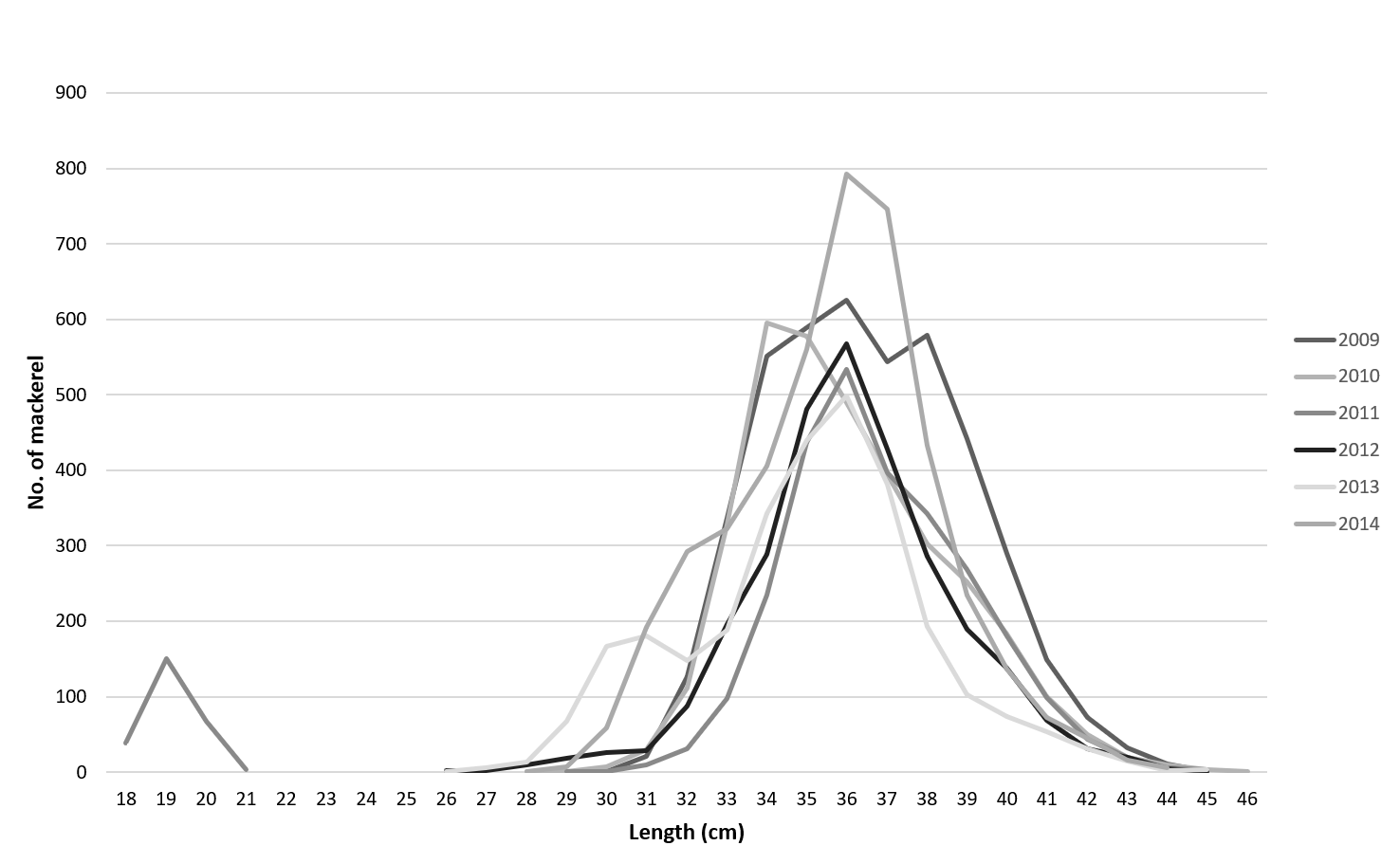

Supplement: S1 Fig — (TIF) [file pone.0225552.s004.tif]
